# Cortical maturation and myelination in healthy toddlers and young children

**DOI:** 10.1016/j.neuroimage.2015.04.058

**Published:** 2015-07-15

**Authors:** Sean C.L. Deoni, Douglas C. Dean, Justin Remer, Holly Dirks, Jonathan O’Muircheartaigh

**Affiliations:** a Department of Pediatric Radiology, Children's Hospital Colorado, Aurora, CO, 80045, USA; b Department of Radiology, University of Colorado Denver, Aurora, CO, 80045, USA; c Advanced Baby Imaging Lab, Brown University School of Engineering, Providence, RI, 02912, USA; d Waisman Laboratory for Brain Imaging and Behavior, University of Wisconsin-Madison, Madison, WI, 53705, USA; e Department of Neuroimaging, King's College London, Institute of Psychiatry, London SE5 8AF, United Kingdom

**Keywords:** Infant brain development, Brain MRI, Myelination, Cortical development

## Abstract

The maturation of cortical structures, and the establishment of their connectivity, are critical neurodevelopmental processes that support and enable cognitive and behavioral functioning. Measures of cortical development, including thickness, curvature, and gyrification have been extensively studied in older children, adolescents, and adults, revealing regional associations with cognitive performance, and alterations with disease or pathology. In addition to these gross morphometric measures, increased attention has recently focused on quantifying more specific indices of cortical structure, in particular intracortical myelination, and their relationship to cognitive skills, including IQ, executive functioning, and language performance. Here we analyze the progression of cortical myelination across early childhood, from 1 to 6 years of age, *in vivo* for the first time. Using two quantitative imaging techniques, namely T_1_ relaxation time and myelin water fraction (MWF) imaging, we characterize myelination throughout the cortex, examine developmental trends, and investigate hemispheric and gender-based differences. We present a pattern of cortical myelination that broadly mirrors established histological timelines, with somatosensory, motor and visual cortices myelinating by 1 year of age; and frontal and temporal cortices exhibiting more protracted myelination. Developmental trajectories, defined by logarithmic functions (increasing for MWF, decreasing for T_1_), were characterized for each of 68 cortical regions. Comparisons of trajectories between hemispheres and gender revealed no significant differences. Results illustrate the ability to quantitatively map cortical myelination throughout early neurodevelopment, and may provide an important new tool for investigating typical and atypical development.

## Highlights

• The maturation of the cerebral cortex is a fundamental process of neurodevelopment.
• We examine the myelination of cortical gray matter in a large cohort of children.
• We observe a logarithmically increasing trend between MWF and age across the cortex.
• Results qualitatively agree with the histological pattern of cortical myelination.
• We find a significant correlation between T1 and MWF throughout the cortex.

## Introduction

Cortical maturation is an important feature of neurodevelopment, characterized by both morphometric and microstructural changes. While alterations in cortical morphological quantities, including volume, thickness, surface area, and curvature, have been associated with neuropsychiatric, neurological, and developmental disorders (for example, Ecker et al., 2009, 2014; Li et al., 2014; Pacheco et al., 2014; Sierra et al., 2014), differences in the rate of development of these quantities across childhood have also been associated with cognitive ability and intelligence (Shaw et al., 2006, 2008). Beyond these gross morphological measures, interest has also focused on exploring the underlying cortical cyto- and myeloarchitecture. Using non-invasive imaging, in particular magnetic resonance imaging (MRI), advances have been made in investigating these attributes *in vivo* through quantitative imaging (voxel-wise mapping of the longitudinal or transverse relaxation times, T_1_ and T_2_, respectively) (Clark et al., 1992) or, more recently, the semi-quantitative ratio of T_1_ weighted (T_1_w) to T_2_ weighted (T_2_w) signal intensity (Glasser and Van Essen, 2011). These methods provide information reflective of the cortical myeloarchitecture, and have been used to identify prominent myeloarchitectural features (i.e., the Stria of Gennari (Clark et al., 1992)), as well as detect boundaries of differentiated cortical regions (Glasser and Van Essen, 2011).

Though myelin is most commonly associated with the brain's white matter, the cortical gray matter also contains significant numbers of myelinated fibers. Like the maturation of the myelinated white matter, myelination of the cortex likely plays a fundamental role in neurodevelopment, and the evolution of cognitive and behavioral functions. Indeed, changes in the T_1_w/T_2_w ratio have been demonstrated across the lifespan (Grydeland et al., 2013; Shafee et al., 2014) from childhood to late adulthood, as well as being associated with cognitive performance (Grydeland et al., 2013).

However, while these prior studies have provided new insight into the evolution of cortical myeloarchitecture throughout older childhood and adulthood, little is known about how this process advances throughout early childhood (from birth through 8 years of age) *in vivo* beyond prior post-mortem investigations (Yakovlev and Lecours, 1967; Brody et al., 1987). Further, while the T_1_w/T_2_w ratio is an easily accessible measure, it is sensitive to both non-myelin tissue changes (i.e., iron and water content) and extrinsic (e.g., imaging hardware, pulse sequence, patient positioning, and post-processing) effects. One of the most established magnetic resonance imaging approaches for investigating myelin content is myelin water fraction (MWF) imaging (MacKay et al., 1994; Laule et al., 2008). Though MWF imaging has previously been used to examine white matter development throughout infancy and childhood (Deoni et al., 2012; O'Muircheartaigh et al., 2014; Dean et al., 2014c), its application to studying cortical development remains unapproached. Therefore, we sought to examine the use of quantitative MWF imaging to investigate cortical myelination in a large cohort of 215 healthy and typically developing toddlers and young children, 1 to 6 years of age. We further compared the MWF of these results to quantitative T_1_ values, which has formed the basis of many prior studies of cortical myeloarchitecture.

## Materials and methods

### Participants

A total of 215 healthy and typically developing children (105 female), 363 to 2198 days (approximately 1 to 6 years) of age, corrected to a 40 week gestation, were recruited from the local surrounding community for this study. Inclusion criteria included: 1. healthy singleton birth between 37 and 42 weeks gestation; 2. uncomplicated pregnancy and delivery; 3. APGAR scores > 8; 4. No reported abnormalities on fetal ultrasound; 5. no reported neurological history in the child; 6. no reported sibling or parental psychiatric history. Full demographic information is provided in Table 1. Inclusion criteria were confirmed during phone interview prior to enrollment, and again through infant and family medical histories taken as part of the study. Maternal SES was determined using the Hollingshead four factor index (Hollingshead, 1975). Written informed consent was obtained from each child's parents or legal guardian, and the study was performed with approval from the host institution's internal review board.

### Data acquisition

Voxel-wise T_1_ and mcDESPOT MWF data were acquired on each child using age optimized DESPOT1 (Deoni et al., 2003, 2005) and mcDESPOT (Deoni et al., 2008, 2013) imaging protocols (Table 2). A constant voxel dimension of (1.8 × 1.8 × 1.8) mm^3^ was used for all children, with the field of view and imaging matrix adjusted depending on child age and head size. The theoretical basis for DESPOT1 and mcDESPOT has been presented in detail previously (Deoni et al., 2003, 2008, 2013) and involves the acquisition of multi-flip angle T_1_-weighted SPGR images (DESPOT1), as well as T_1_/T_2_-weighted bSSFP images at two differing radio-frequency (RF) phase-cycling patterns (Deoni, 2011). To these data, conventional single-component or multicomponent relexometry may be performed. For multicomponent analysis, a 3-pool tissue model comprising of myelin-associated, intra and extra cellular, and CSF water pools is fit to the combined SPGR and bSSFP data using an iterative stochastic region approach (Deoni and Kolind, 2015). Correction for flip angle (transmit magnetic field) inhomogeneities is performed through additional acquisition of inversion-prepared (IR-)SPGR data, and the two phase-cycled bSSFP data allows correction from main magnetic field variations (Deoni, 2011).

In addition to the quantitative T_1_, T_2_ and MWF data, a higher-resolution (1.2 × 1.2 × 1.2) mm^3^ T_1_-weighted anatomical IR-SPGR image was also acquired (Table 2). As above, the imaging FOV and matrix size were adjusted to maintain the same voxel dimensions over the full head volume.

All children were imaged on a Siemens Tim Trio 3 T scanner. Scanning was performed during natural, *non-sedated* sleep, or, if tolerated, while watching a favorite movie. To minimize acoustic noise, the maximum imaging gradient slew rates and peak values were reduced. Passive measures, including a sound-insulating bore liner, MiniMuff ear pads, and sound-attenuating ear protectors were also employed (Dean et al., 2014a).

### Cognitive and behavioral assessment

To ensure that children included in this study were typically developing and had average cognitive ability for age, each child was assessed following MRI using the Mullen Scales of Early Learning (Mullen, 1995). These scales provide age-normalized scores for fine and gross motor control, visual reception, and expressive and receptive language for children up to 5 years, 9 months of age, as well as an overall Early Learning Composite, ELC, expressed as a standard score with mean 100 and standard deviation of 15. In addition, assessments including the Modified Checklist for Autism in Toddlers (M-CHAT) (Chlebowski et al., 2013) and the Communication and Symbolic Behavior Scales Developmental Profile (CSBS-DP) (Wetherby and Prizant, 2002) were used to identify children with abnormal behaviors or language delay. No children included in this study screened as at-risk using these broad assessment tools or had an ELC < 85 (i.e., less than one standard deviation below the mean).

### Image analysis

Following image acquisition, the mcDESPOT data from each child were linear co-registered to account for subtle intra-scan motion (Jenkinson et al., 2002), non-brain signal was removed (Smith, 2002), B_0_ and B_1_ field calibration maps were calculated (Deoni, 2011), and voxel-wise T_1_ and MWF maps were calculated (Deoni et al., 2013).

### Surface projections of cortical T_1_ and MWF

The higher resolution anatomical image for each child was intensity normalized using the N3 algorithm (Boyes et al., 2008; Zheng et al., 2009) and the Freesurfer analysis pipeline (Fischl, 2012) was used to segment the cortical ribbon and delineate the cortex into 34 regions per hemisphere. At each stage throughout the processing pipeline, images were visually inspected and, if needed, manually edited and corrected. This included inspecting data for poor skull-stripping, which required re-running this processing step, the additional use of gcut (http://freesurfer.net/fswiki/FsTutorial/SkullStripFix_freeview) and, in extreme cases, manual removal of the remaining dura and eye signal. Additional editing was required on each of the participants under 3 years of age.

The calculated T_1_ and MWF maps from each participant were then linearly co-registered to the corresponding high resolution anatomical image by first aligning the high flip angle T_1_-weighted SPGR image acquired as part of the mcDESPOT protocol, and then applying the calculated transformation matrix to the quantitative T_1_ and MWF images. At each point along the cortical surface, the T_1_ and MWF value at the halfway depth along the surface normal was obtained and projected onto the surface. Sampling at the midpoint of the cortical surface was chosen to ensure values were from within the cortical ribbon and not the underlying adjacent white matter. This process was repeated for each of the 215 children. Age-averaged T_1_ and MWF surfaces were then calculated by registering all children's surfaces to a custom Freesurfer template, and averaging children between: 363–712 days (mean = 547 ± 112, *n* = 78); 718–1099 days (mean = 931 ± 116, *n* = 55); 1102–1442 days (mean = 1278 ± 98, *n* = 32); 1469–1814 days (mean = 1700 ± 110, *n* = 29); and 1836–2198 days (mean = 2022 ± 118, *n* = 21). The custom template was created using the make_average_subject command within Freesurfer (http://surfer.nmr.mgh.harvard.edu/fswiki/make_average_subject) and including 5 male and 5 age-matched females from each age-range (for a total of 50 participants). This was done so as to not bias the template to any particular gender or age range. Age ranges were chosen so as to end up with group mean ages of 1.5, 2.5, 3.5, 4,5, and 5.5 years.

### Developmental trajectories of cortical T_1_ and MWF

To visualize and investigate how cortical T_1_ and MWF change with age, we next superimposed each of the 68 delineated cortical regions (34 per hemisphere) for each child onto their T_1_ and MWF maps, and calculated the mean and standard deviation regional T_1_ and MWF. These mean values were then plotted against the child's age, corrected to a 40-week gestation. To these plots, we then fit continuous linear, quadratic, and logarithmic functions and compared Bayesian information criterion (BIC) values for each to determine which provided the most parsimonious fit to the data.

### Hemispheric asymmetry in cortical T_1_ and MWF

To examine potential hemispheric differences or asymmetries in regional cortical T_1_ and MWF development, we first plotted each cortical region's cross-sectional T_1_ and MWF values, separated by hemisphere. Logarithmic curves were then fit to each hemisphere's data, as well as to the combined left + right hemisphere data. An *F*-test was then used to determine if the data justified the fitting of two hemisphere-specific curves. If a region passed this *F*-test step, a bootstrap with residual sampling approach was used to reconstruct the logarithmic slope and intercept distributions for each hemisphere, and these distributions were then compared using an unpaired *t*-test. Significance was defined as *p* < 0.0014 (*p* < 0.05 corrected for the 34 region comparisons).

In addition, we also calculated the mean index of laterality (left − right / left + right) for each region and for each individual. These values were then plotted against the individual's age. A single sample *t*-test was used to determine if the laterality index differed significantly from 0 (fully symmetric) across the entire cohort age range. We examined the effect of age on laterality more specifically by using a sliding window approach. Here, the mean laterality index was calculated, and a single population *t*-test was performed, using a constant window of 50 subjects. That is, ordering subjects by increasing age, the first 50 children (#1–#50) were grouped and the laterality index calculated; then subjects #2–#51 were grouped and laterality index calculated, and so forth. Significance was defined as *p* < 0.00035 (*p* < 0.05 corrected for the 154 repeated measures per region).

### Gender differences in cortical T_1_ and MWF

As prior studies of cortical thickness development across older childhood and adolescence have observed gender related differences (Sowell et al., 2007), we sought to determine if similar differences could be seen in the cross-sectional trajectories of T_1_ or MWF development in our younger cohort. As with the hemisphere analysis above, we first plotted each cortical region's cross-sectional T_1_ and MWF values for each gender independently. Logarithmic curves were then fit to each gender's data, as well as to the combined male + female data. An *F*-test was then used to determine if the data justified the fitting of gender-specific curves. For regions that passed this step, a bootstrap approach reconstructed the logarithmic slope and intercept distributions for each gender, and these distributions were then compared using an unpaired *t*-test. Significance was defined as *p* < 0.0014 (*p* < 0.05 corrected for the 34 region comparisons).

## Results

### Surface projections of cortical T_1_ and MWF

**Fig. 1:**
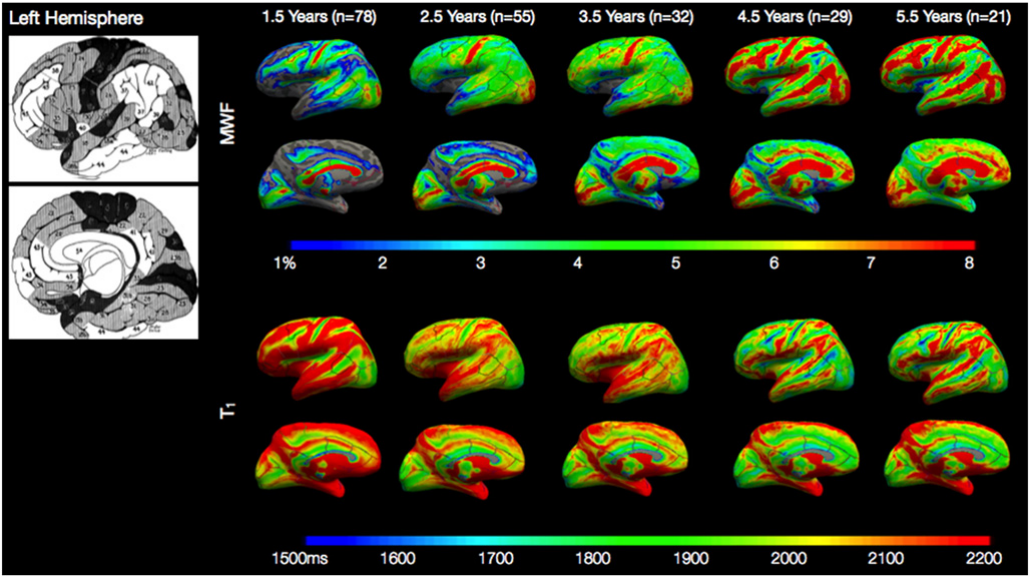
Left hemisphere age-averaged surface projections of cortical MWF and T_1_ relaxation times calculated at 1.5, 2.5, 3.5, 4.5, and 5.5 years of age. Shown for reference is a map of cortical myelination produced by Flecshig (Flechsig, 1901), in which early myelinating regions are depicted in dark shading, and late myelinating regions in white.

**Fig. 2:**
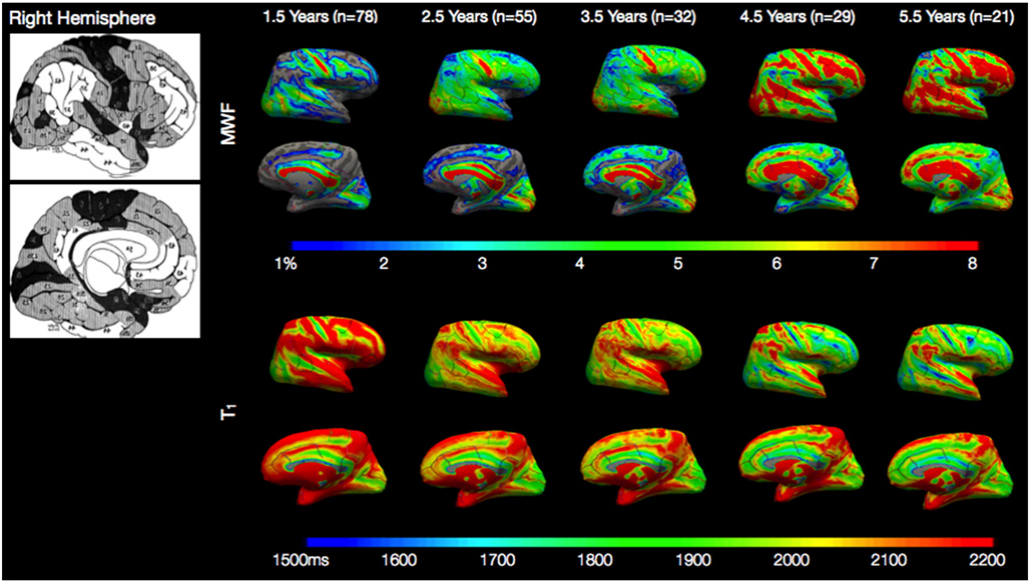
Right hemisphere age-averaged surface projections of cortical MWF and T_1_ relaxation times calculated at 1.5, 2.5, 3.5, 4.5, and 5.5 years of age. Shown for reference is a map of cortical myelination produced by Flecshig (Flechsig, 1901), in which early myelinating regions are depicted in dark shading, and late myelinating regions in white.

Figs. 1 and 2 show the average cortical surface projections of MWF and T_1_ for each of the five age-groups: 1.5 years (363–712 days); 2.5 years (718–1099 days); 3.5 years (1102–1442 days); 4.5 years (1469–1814 days); and 5.5 years (1836–2198 days). Examining these maps we see the expected trend of increasing MWF, and decreasing T_1_, across age. Examining the earliest map (1.5 years), we note a strong qualitative similarity between regions of higher and lower MWF and regions depicted by Flechsig and others as myelinating earlier or later during development (Flechsig, 1901). Specifically, the primary somatosensory and motor, primary and secondary visual, and auditory cortices; and superior temporal gyrus are seen to have high MWF (and low T_1_) bilaterally at 1.5 years of age. In contrast, areas such as the temporal pole, prefrontal and association cortex have low MWF (and high T_1_) at this age. These regions map onto those areas histologically shown to have rapid or protracted myelination, respectively.

**Fig. 3:**
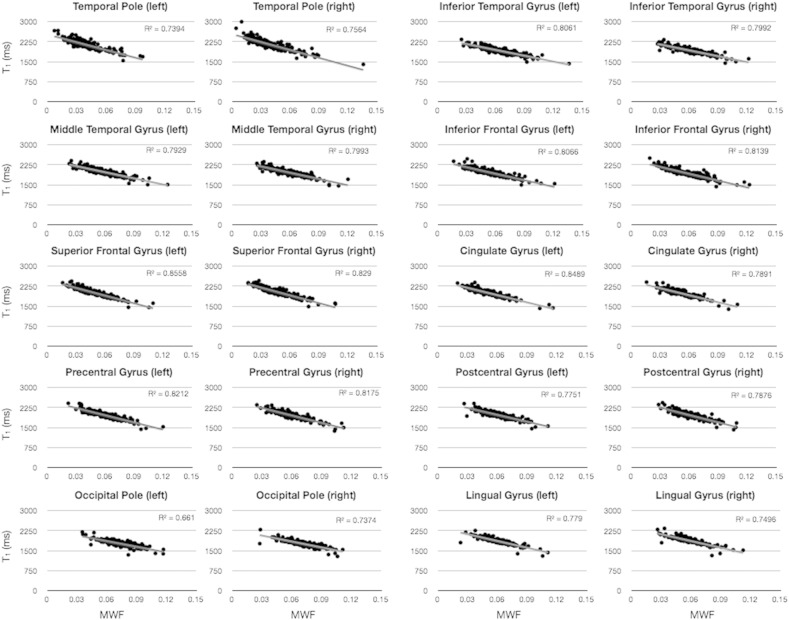
Plots of the relationship between T_1_ and MWF in each cortical region. In all investigated regions, we found a significant negative relationship, with greater MWF predicting decreased T_1_.

As the T_1_ relaxation time is related to lipid myelin and macromolecule content (in addition to water content and iron content), we examined the relationship between T_1_ and MWF in each cortical region. In Fig. 3, we show a subset of the 34 bilateral regions, composed of early, middle, and late myelinating regions as illustrated in Figs. 1 and 2. In all cases, T_1_ and MWF were found to be significantly correlated, with correlation coefficients *R* > 0.75 in all investigated regions. This result in cortex differs from similar analysis performed in white matter (Deoni et al., 2012), in which the relationship between MWF and T_1_ was found to be weak, and varies depending on age.

### Developmental trajectories of cortical T_1_ and MWF

**Fig. 4:**
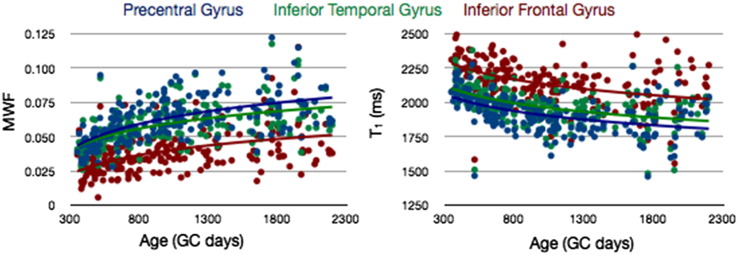
Comparison of MWF vs. age (left) and T_1_ vs. age (right) trajectories calculated for early (precentral gyrus), moderate (inferior temporal gyrus), and late (inferior frontal gyrus) myelination regions.

To investigate how cortical T_1_ and MWF change with age, a series of growth models, including linear, logarithmic, and quadratic functions were fit to the mean values, and the models were compared using the BIC. Results of this analysis (Table 3) show that both T_1_ and MWF across the investigated age range are best characterized by logarithmic functions. As a result of this analysis, for all following analysis and results, logarithmic functions were used to model the growth trajectories. In Fig. 4, a comparison of T_1_ and MWF vs. age for a selection of early and fast (precentral gyrus), moderate (inferior temporal gyrus), and late and slowly (inferior frontal gyrus) maturing brain regions is shown.

### Hemispheric asymmetries

**Fig. 5:**
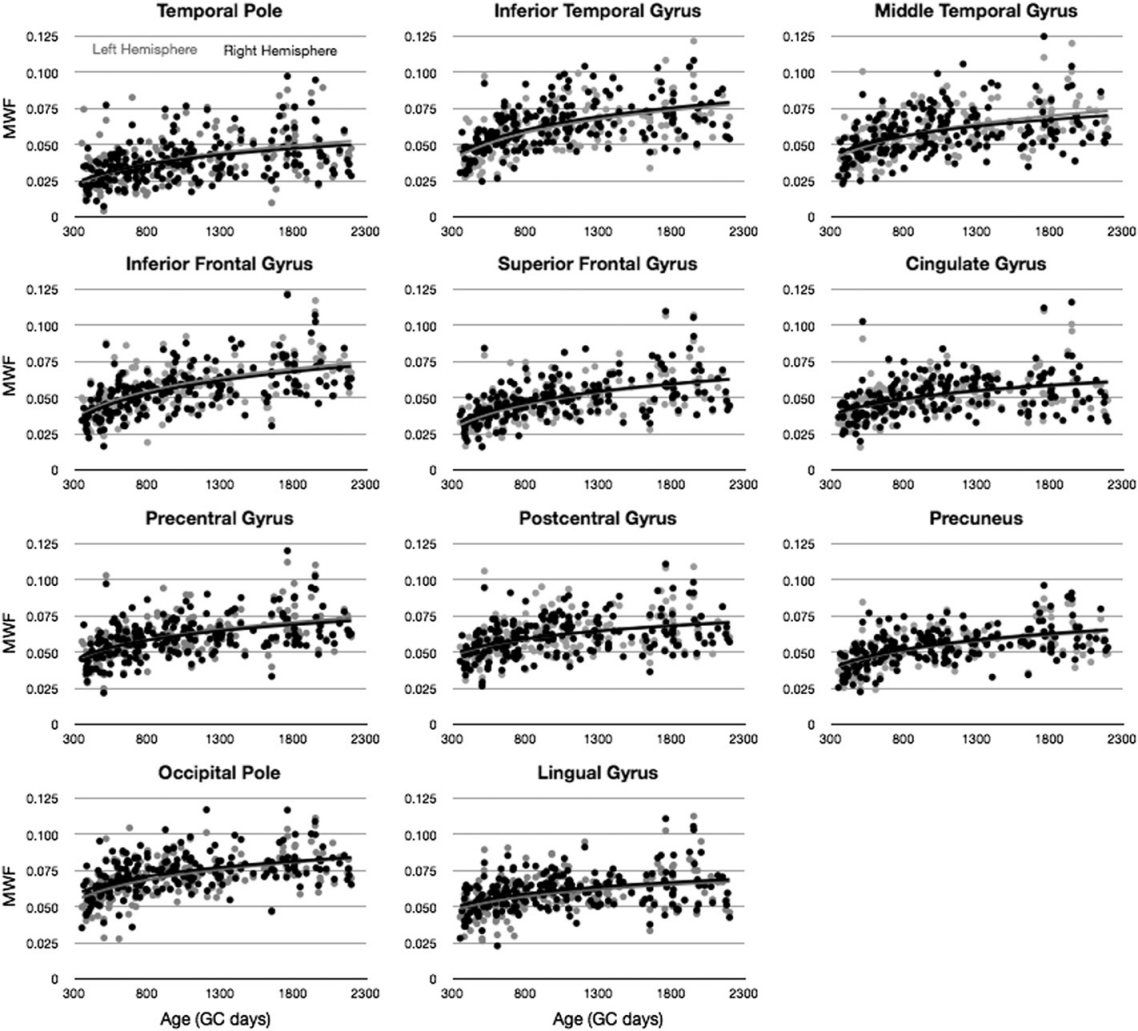
Comparison of MWF vs. age trajectories, divided by hemisphere, for a selection of cortical regions. Light gray points and line correspond to left hemisphere. Points correspond to the mean MWF value calculated within the region, and the line corresponds to the logarithmic trend line.

**Fig. 6:**
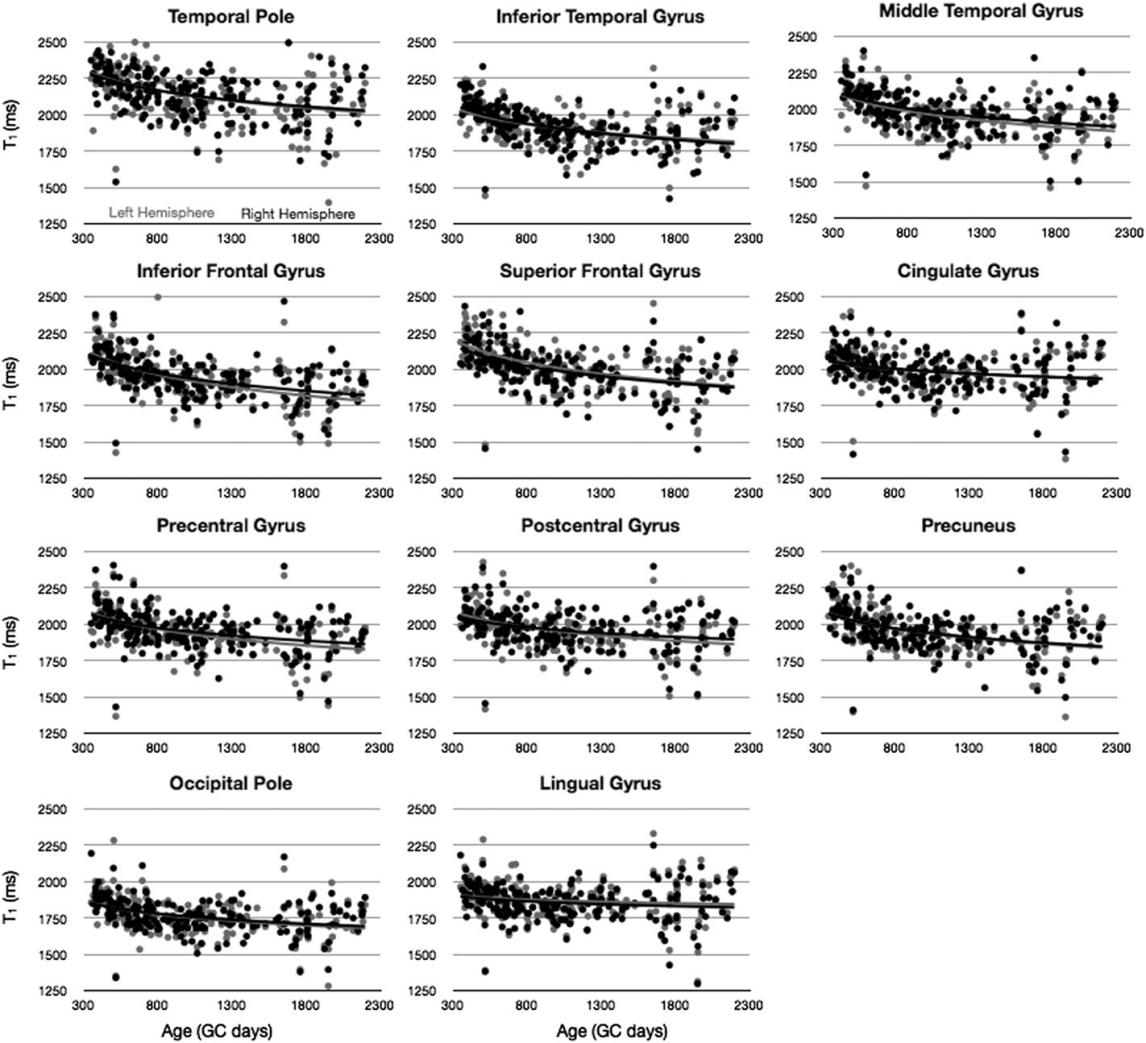
Comparison of T_1_ vs. age trajectories, divided by hemisphere, for a selection of cortical regions. Light gray points and line correspond to left hemisphere. Points correspond to the mean MWF value calculated within the region, and the line corresponds to the logarithmic trend line.

Building on the results presented in Table 3 and Fig. 4, we investigated potential hemispheric differences and laterality in the cross-sectional developmental trajectories of each cortical region. In Figs. 5 and 6, MWF and T_1_ vs. age trajectories for 11 representative regions are shown, color-coded by hemisphere. An *F*-test performed on each region revealed that no region justified fitting each hemisphere independently for either MWF or T_1_. Thus, there were no differences in the rate of development between the left and right hemisphere any of the 34 regions.

**Fig. 7:**
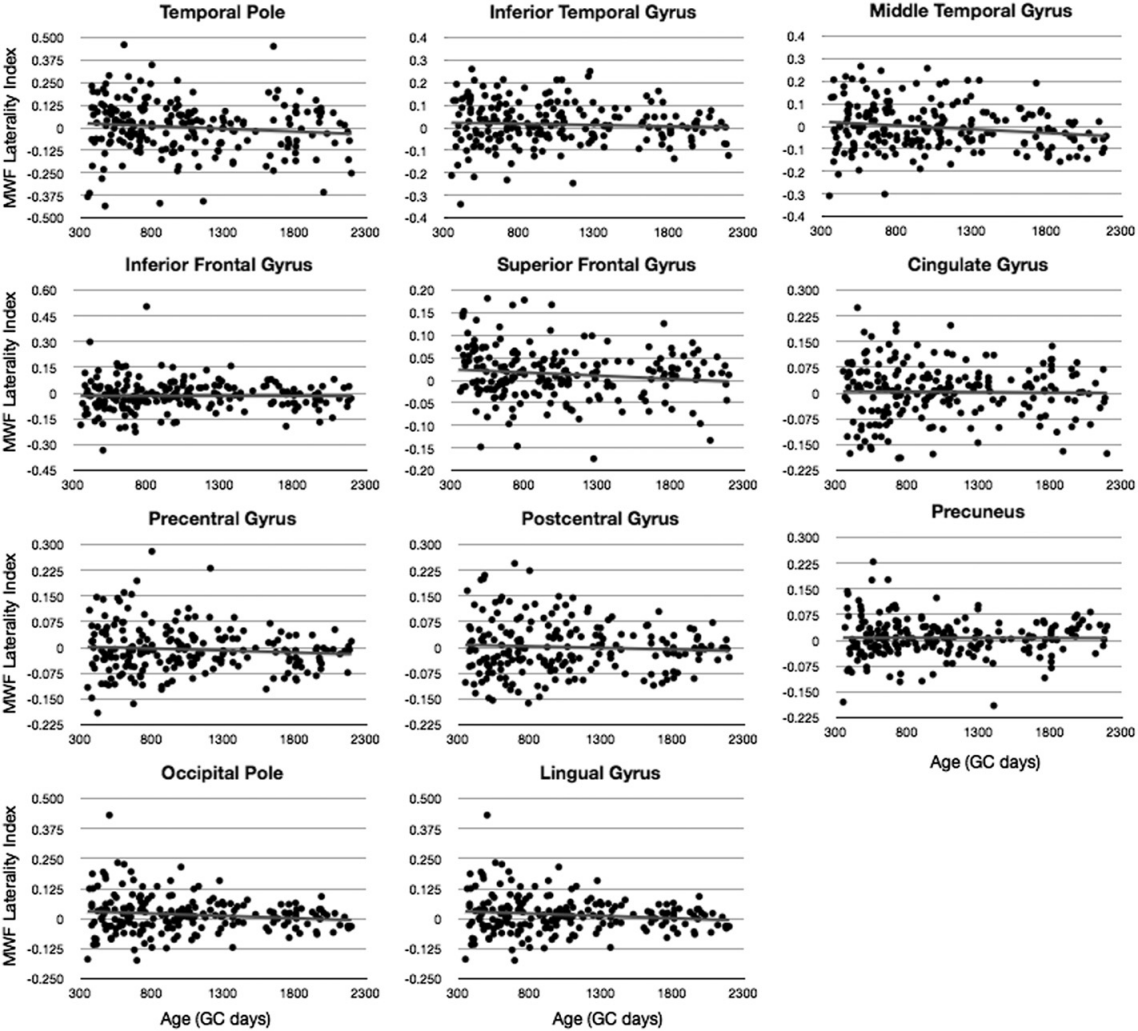
Analysis of hemispheric MWF asymmetry. Plots correspond to MWF laterality index (left MWF − right MWF / left MWF + right MWF) calculated for each individual.

**Fig. 8:**
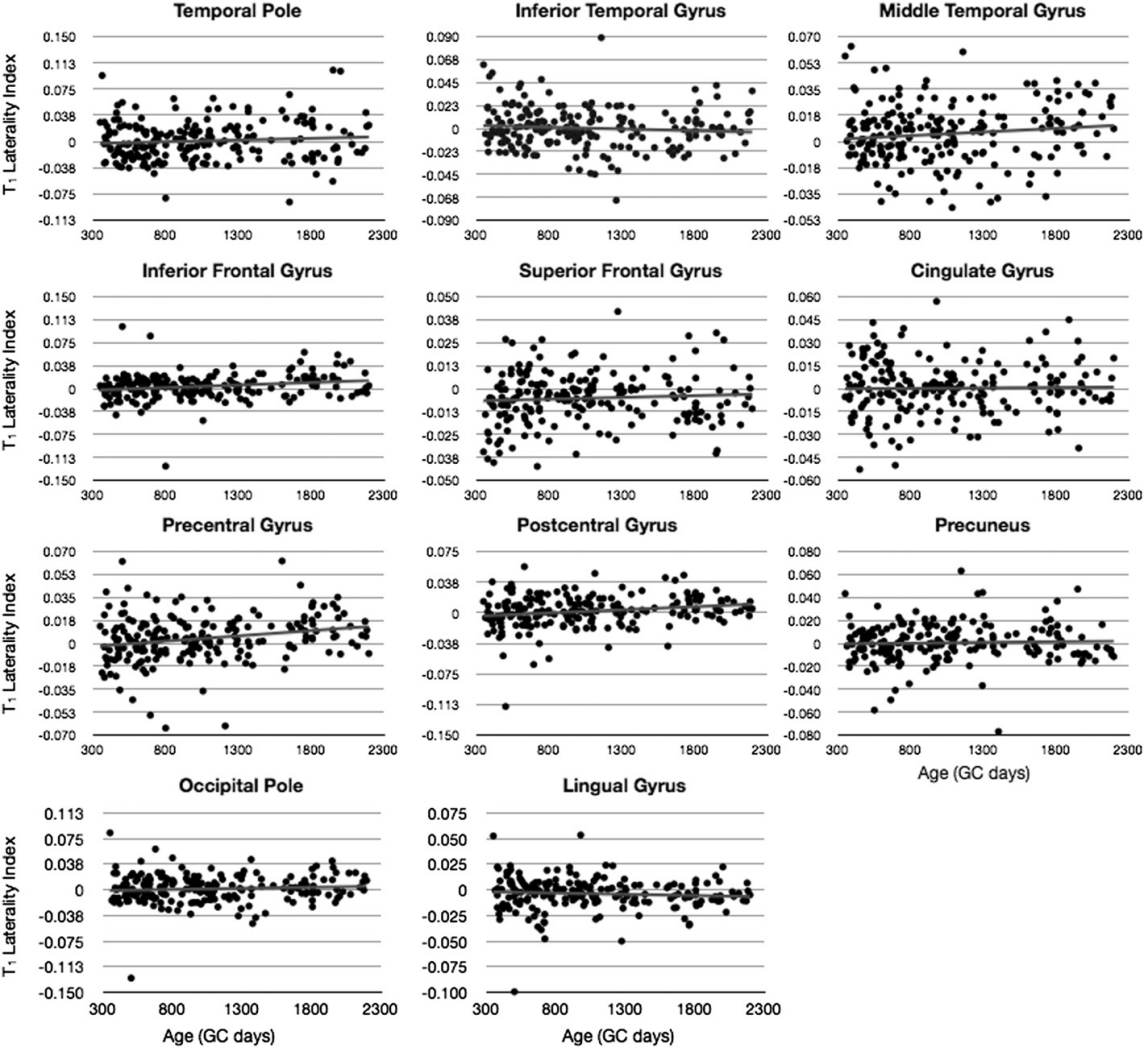
Analysis of hemispheric T_1_ asymmetry. Plots correspond to T_1_ laterality index (left T_1_ − right T_1_ / left T_1_ + right T_1_) calculated for each individual.

To determine if there was a significant left–right hemisphere difference in MWF or T_1_ magnitude, we quantified and examined the laterality index, LI (Figs. 7 and 8). Performing a single sample *t*-test to determine if the mean LI differed significantly from 0, we found that the occipital pole, lingual gyrus, and superior frontal gyrus were significantly left lateralized (LI > 0) with respect to MWF after corrected for the 34 individual comparisons. With respect to T_1_, lingual gyrus and superior frontal gyrus, were found to be significantly right lateralized (LI < 0, *p* < 0.05 corrected), in agreement with the relationships shown in Fig. 3, with increased T_1_ predicting decreased MWF.

**Fig. 9:**
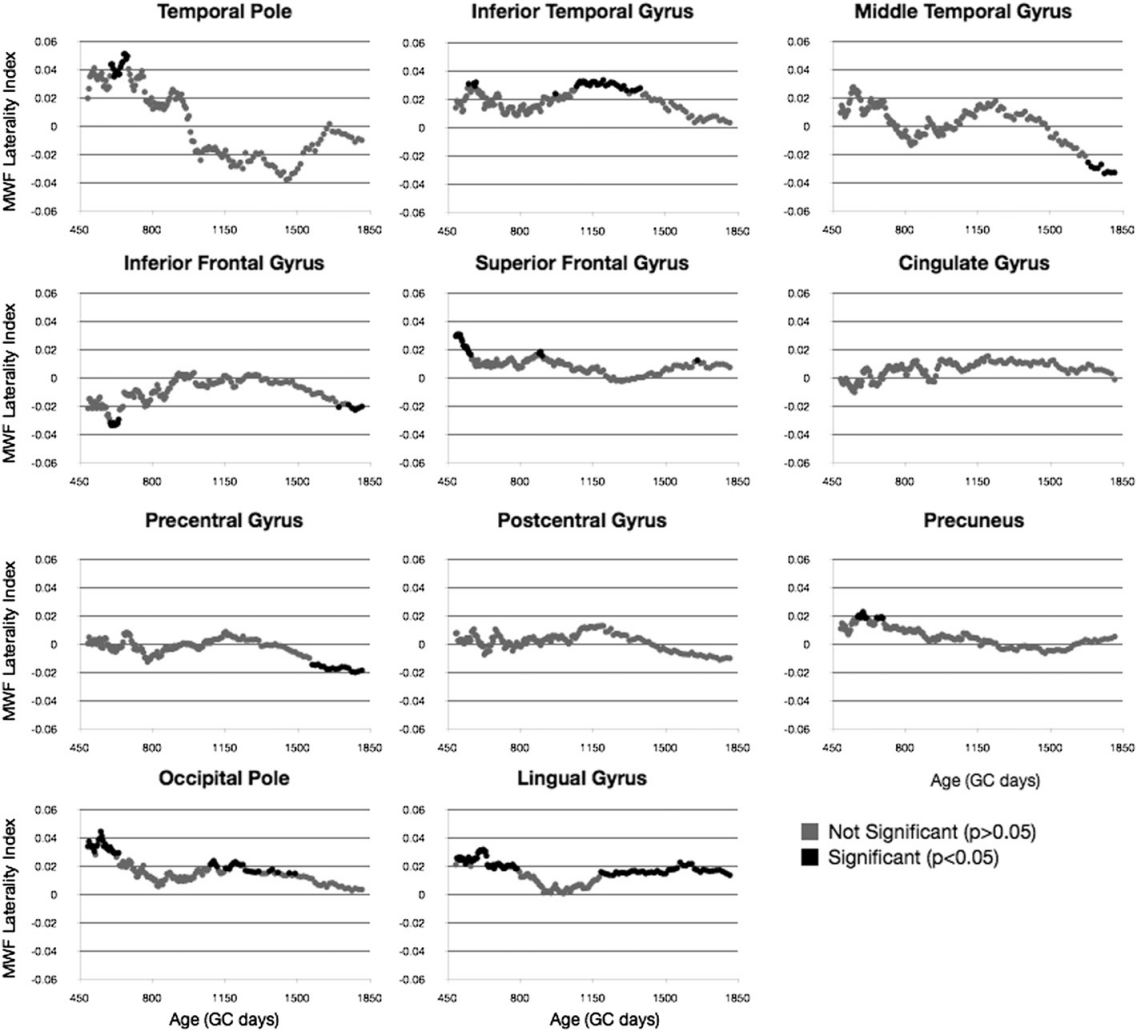
Sliding window analysis of MWF asymmetry. The mean laterality index was calculated for a sliding and overlapping window of 50 children and plotted with respect to the mean age for the window. A single sample *t*-test was used to determine if the calculated mean index differed significantly from 0. Areas that reached significance (*p* < 0.05, corrected for the 132 comparisons per region) are denoted by dark shading.

**Fig. 10:**
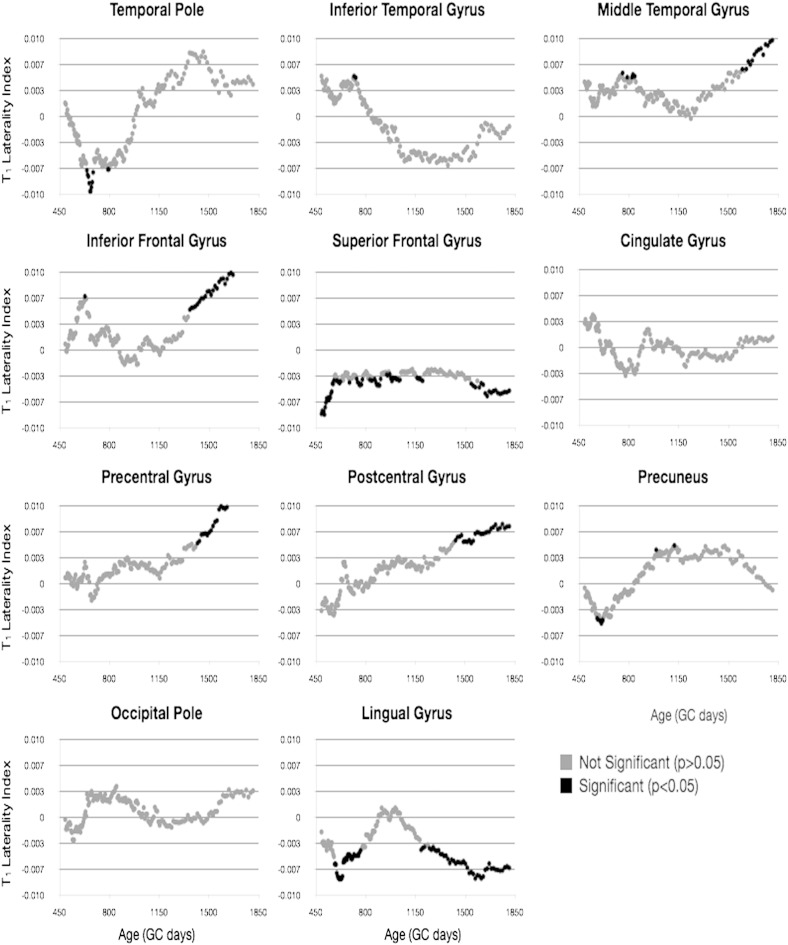
Sliding window analysis of T_1_ asymmetry. The mean laterality index was calculated for a sliding and overlapping window of 50 children and plotted with respect to the mean age for the window. A single sample *t*-test was used to determine if the calculated mean index differed significantly from 0. Areas that reached significance (*p* < 0.05, corrected for the 132 comparisons per region) are denoted by dark shading.

Finally, using a sliding window approach, we examined age-related changes in LI by testing the mean LI against 0 in blocks of 50 children. Results of this analysis (Figs. 9 and 10) show differential age-related trends depending on cortical region. Regions including the temporal pole, inferior temporal gyrus, inferior and superior frontal gyrus, precuneus, occipital pole, and lingual gyrus show significant leftwards laterality (except for inferior frontal, which had significant rightward laterality) in early childhood, prior to 2 years of age. Regions including the inferior temporal gyrus, occipital pole, and lingual gyrus exhibit significant leftwards asymmetry between 3 and 4 years of age. And regions, including precentral gyrus and lingual gyrus, show significant right or left asymmetry, respectively, in late childhood (i.e., 4.5 years of age and older).

### Sexual dimorphism

**Fig. 11:**
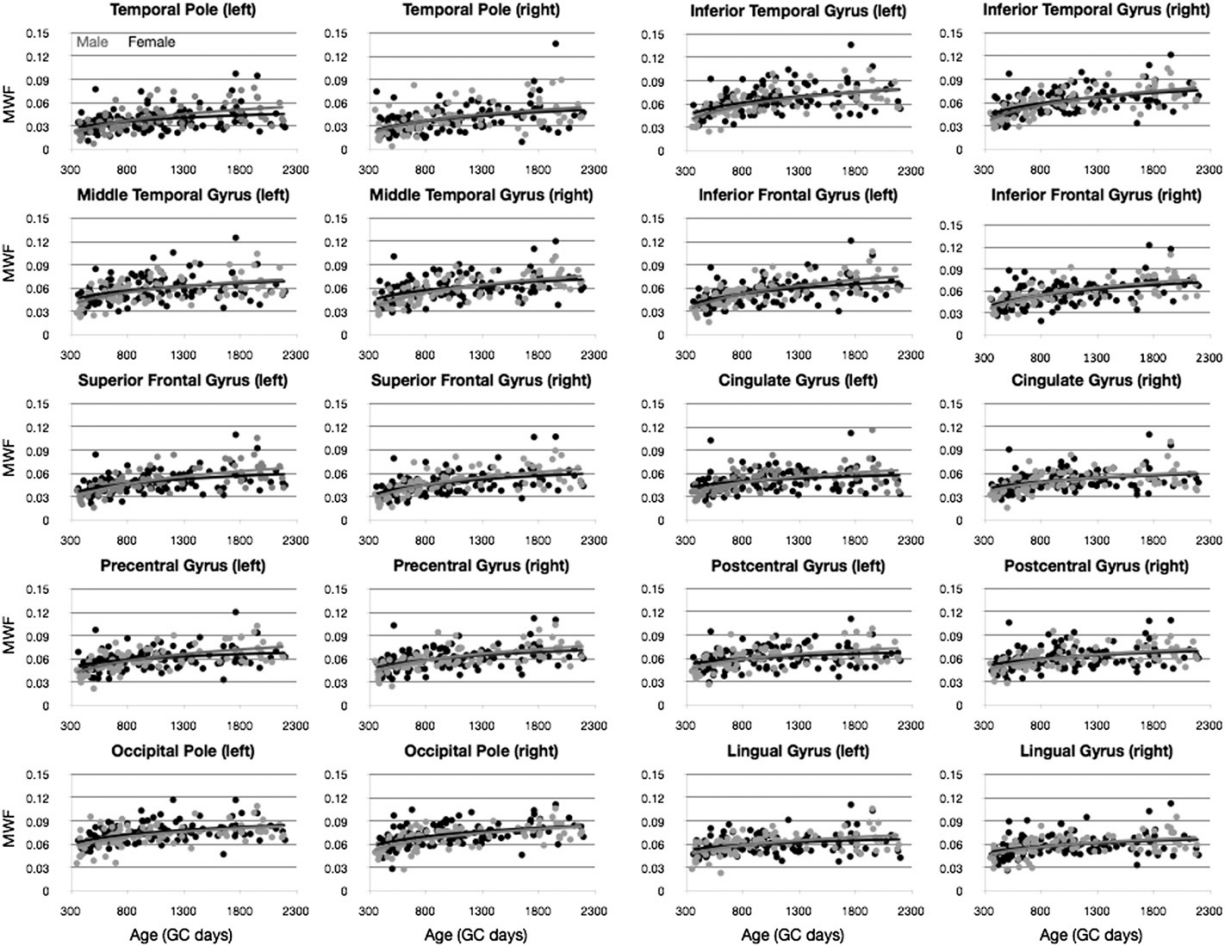
Comparison of MWF vs. age development trajectories divided by gender. Light gray points and line correspond to male participants. Points correspond to the mean MWF value calculated within the region, and the line corresponds to the logarithmic trend line. No significant differences were identified.

**Fig. 12:**
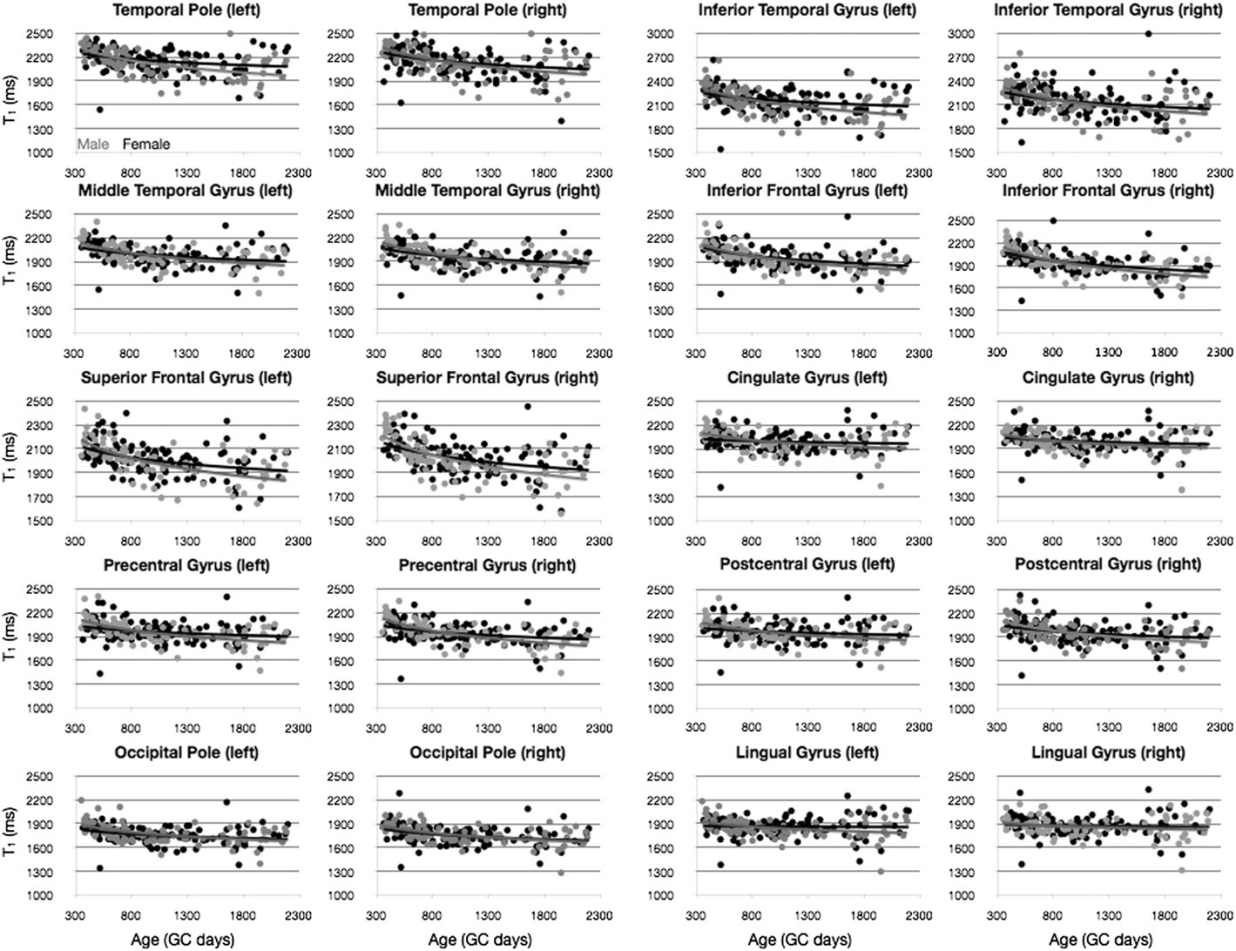
Comparison of T_1_ vs. age development trajectories divided by gender. Light gray points and line correspond to male participants. Points correspond to the mean MWF value calculated within the region, and the line corresponds to the logarithmic trend line. No significant differences were identified.

Finally, we examined differences in the development if MWF and T_1_ vs. age in children stratified by gender (Figs. 11 and 12). As above, we first used an *F*-test to determine if the data from each cortical region justified the fitting of each gender separately, and found no region in which this was supported. Further, an unpaired *t*-test was used to determine if there was a significant male–female difference in mean MWF or T_1_ across the age-range. No significant differences were found, even without correcting for multiple comparisons.

## Discussion

Alongside white matter maturation, cortical development plays an important role in the development of cognitive and behavioral functioning. In this work, we have sought to extend our prior investigations of white matter maturation by examining cortical myelination throughout early childhood for the first time using quantitative T_1_ and MWF imaging. Results of this analysis reveal a pattern of cortical myelination consistent with that previously established histologically using postmortem brain specimens. That is, myelination appears earliest in primary somatosensory, motor, visual, and auditory cortices, whereas areas including the temporal pole, prefrontal and association cortices are among the last to mature. Consideration of prior reports of cortical gray matter volume growth in early childhood (Gilmore et al., 2012) suggests a qualitative relationship between myelination and the rate of gray matter volume growth. Specifically, areas with early myelination (high myelin content by 1 year of age), e.g., motor and visual cortices, appear to have fast volume growth between birth and 1 year of age, but slower growth between 1 and 2 years of age. In contrast, cortical regions with later myelination (e.g., temporal and angular gyri), exhibit slow growth between birth and 1 year of age, but rapid gray matter growth between 1 and 2 years of age. As myelin constitutes a significant portion of brain volume (Zabin, 1956), it is likely that the increase of cortical myelin is a primary driver of cortical gray matter volume growth. However, this hypothesized relationship may only be valid during the earliest stages of neurodevelopment. In prior investigations of cortical thinning during later childhood and adolescence (Sowell et al., 2004), the authors proposed that observed cortical thinning may be the result of increased myelination of neural fibers in the lower cortical layers.

**Fig. 13:**
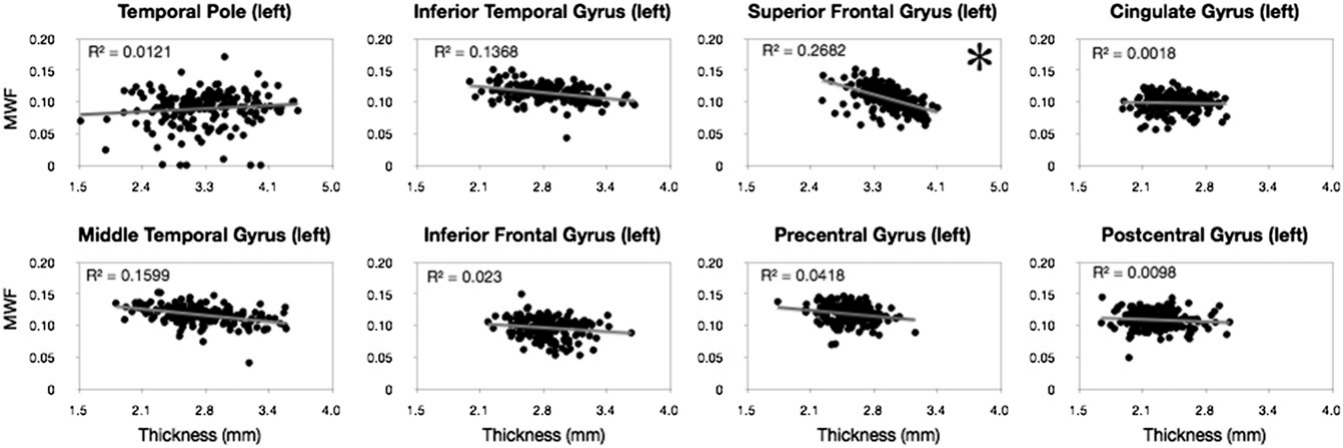
Ad-hoc preliminary analysis investigating the relationship between cortical MWF and thickness. Only the superior frontal gyrus (both left and right) were found to have a significant negative relationship (denoted by the asterisk). This result may suggest that cortical volume growth and cortical myelination may be independent processes.

To examine the relationship between cortical MWF and thickness, we performed an ad-hoc investigation comparing these values in the cortical region. A summary of results of this analysis, shown in Fig. 13, presents a more complex picture. While some regions, such as the superior frontal gyrus exhibit a significant (*p* < 0.05 FWE corrected) *negative* relationship between cortical thickness and MWF (with increased MWF predicting small cortical thickness), most regions showed a non-significant relationship. While more detailed analysis is required, particularly to examine how early MWF changes may predict later thickness changes (or vice versa), these preliminary results may suggest that cortical myelination and growth are independent processes.

Within each of the cortical regions examined, MWF was found to increase, and T_1_ to decrease, logarithmically from 1 to 6 years of age. These developmental trends broadly parallel the trajectories noted in white matter across the same age range (Deoni et al., 2012; Dean et al., 2014b), and are predictive of later age trajectories. T_1_ has been shown to decrease from childhood until approximately 40 years of age, depending on brain region (Steen et al., 1995; Cho et al., 1997). Similarly, recent analysis using the T_1_w/T_2_w ratio from 8 to 83 years of age noted an ‘inverted U’ trajectory (Grydeland et al., 2013; Shafee et al., 2014), with cortical myelin increasing until approximately 40 years of age, and then decreasing with advancing age.

Across the investigated age range, and in each cortical region, we found that MWF and T_1_ were significantly correlated. While changes in T_1_ have previously been linked to changing myelin content in the human brain, evident from both high resolution investigations of cortical myeloarchitecture (Clark et al., 1992; Sereno et al., 2013), as well as changes in the T_1_w signal during neurodevelopment (Barkovich et al., 1988; Paus et al., 2001), T_1_ is also influenced by a myriad of other factors, including iron and water content (Bottomley et al., 1984; Gelman et al., 2001). Thus, the demonstrated relationship between T_1_ and MWF suggests that changes in myelin content may be the dominant influence on cortical T_1_ in early development. Further, quantitative T_1_ may be an acceptable surrogate marker of myelin content within the cortex during this period. However, caution is urged in assuming this relationship in atypical development or disease, in which altered tissue microstructure, edema, or lipid and protein content could also alter T_1_ (Bottomley et al., 1987). Of note, this result differs from our own prior results in cerebral white matter, which showed no significant relationship between T_1_ and MWF (Deoni et al., 2012). This seemingly contradictory result could be further evidence of the notoriously non-specific nature of T_1_, which is influenced by water content, iron content, fiber density, macromolecule and lipid content, etc. The maturation of white matter is substantively different than the maturation of cortical gray matter, and the development of the myelin sheath, while the most visibly striking change in MR images, may not be the dominant influence on T_1_.

In addition to developmental trajectories, we also investigated hemispheric asymmetry in MWF and T_1_. In prior investigations of older children (Reiss et al., 1996), as well as fetal brain (Chi et al., 1977), statistically significant left–right asymmetries have been noted in global and regional cortical gray matter volume. Averaged across our full cohort and age range, we found no cortical asymmetry in MWF or T_1_. However, investigating asymmetry as a function of age revealed differential trends, in which some regions showed significant asymmetry at 1 year of age (e.g., superior frontal gyrus, occipital pole) but not at later ages; and other regions displaying significant asymmetry only at older ages (e.g., precentral and lingual gyri). Age-related changes in asymmetry may reflect on-going changes in functional localization, and the consolidation of brain structure–function relationships (Toga and Thompson, 2003). In this work, we did not specifically examine the influence of gender, or handedness on right–left asymmetry (Reiss et al., 1996; Toga and Thompson, 2003; Shaw et al., 2009).

Although gender-related differences have been previously noted in brain development, with boys reported to have increased cortical gray matter volume than girls by 5 years of age (Reiss et al., 1996), we found no significant male–female difference in cortical MWF or T_1_ values. Though significant associations have previously been documented between handedness and regional thickness (asymmetry) measures within the parietal and frontal cortices (Hamilton et al., 2007), we did not specifically address this in our data. This stemmed simply from our inability to reliably assess handedness in the youngest participants (under 18 months of age), and the potential inaccuracy in children between 18 and 30 months, since only 60–70% of children maintain handedness between this age range (Fagard and Lockman, 2005). Following these children further, however, may allow us to not only investigate asymmetries associated with handedness, but from longitudinal data, determine if and when handedness may be predicted.

While this work details the first *in vivo* description of cortical myelination throughout early childhood, there remain some methodological limitations. The primary of these is the relatively low spatial resolution of the acquired MWF and T_1_ images, (1.7 × 1.7 × 1.7) mm^3^, which may introduce partial volume effects that vary depending on child age and cortical region. This is particularly true in regions such as the primary somatosensory and visual cortex, which even in adults may be less than 2 mm thick (Triarhou, 2007). In these regions, the estimated MWF or T_1_ value may be under or over-estimated, depending on whether the region contains CSF of white matter voxel averaging. This effect may explain the low MWF (and high T_1_) values evident in the superior and medial portion of the somatosensory and motor cortices (particularly noticeable at 1.5 years of age) in Figs. 1 and 2. In an attempt to minimize this, we projected MWF and T_1_ values from the middle of the cortical ribbon, rather than choosing values closer to the pial surface or white/gray matter boundary. To fully remove partial volume effects, acquisitions with increased spatial resolutions (ideally less than 1 mm^3^ isotropic) are likely necessary. While the time duration for such acquisitions may be too long for *non-sedated* pediatric imaging, the use of modern multi-channel radio-frequency coils combined with modest parallel imaging acceleration could permit whole-brain 1 mm^3^ isotropic MWF and T_1_ maps in less than 10 min in applications where acoustic noise is less of a concern.

A second concern lies in the application of Freesurfer (Fischl, 2012) in children under 4 years of age. Though few studies have utilized Freesurfer in this young age range, owing to the difference in T_1_w image contrast and potential biases in the cortical parcellation, success has been reported in toddlers as young as 12 months (Travis et al., 2014) — similar to the youngest toddlers included in this study. We purposely excluded children under 1 year of age from this study due to the lack of sufficient gray/white matter contrast that led to inaccurate cortical segmentation. This effect was most pronounced in children less than 6 months of age, but was relatively resolved by 9 months of age. At each stage of analysis, images where checked manually to ensure accurate segmentation. While the spatial resolution of the T_1_w images used for segmentation was less than the ideal 1 mm^3^ isotropic recommended for adult studies, our resolution of (1.2 × 1.2 × 1.2) mm^3^ is in line with, if not higher than, other pediatric imaging studies (Shaw et al., 2011; Shafee et al., 2014).

A final concern lies in the interpretation of mcDESPOT MWF values as specifically representing myelin content. There exists a known bias between mcDESPOT-derived values, and those calculated using more established multi-echo approaches (Zhang et al., in press). Though the reason for this bias remains unknown alternative signal sources, including magnetization transfer (MT), have been suggested as potential confounds, though recent work indicates that MT effects do not significantly affect mcDESPOT values (Zhang et al., in press). The large numbers of independent variables contained within the mcDESPOT signal model may also impart bias or sensitivity to initial estimates; however recent simulation work suggests mcDESPOT MWF values are reproducible across a variety of algorithm choices and experimental conditions (Deoni and Kolind, 2015). While histological verification of mcDESPOT has, to date, been limited to qualitative histological comparisons in the shaking pup model of dysmyelination (Hurley et al., 2010), the specificity of mcDESPOT to myelin can also be garnered indirectly through comparison with the known histological time-course of myelination in human infants (Deoni et al., 2011, 2012), and demyelination studies in MS (Kolind et al., 2012; Kitzler et al., 2012). These animal model and *in vivo* results give confidence that if not specific to myelin, mcDESPOT provides novel information regarding white matter microstructure, and offers differing, perhaps enhanced, sensitivity to myelin changes (Dean et al., 2014b).

The quantitative imaging methods utilized herein provide potential advantages over T_1_w and T_1_w/T_2_w ratio studies. Within the MRI literature, the term “mapping” is traditionally reserved for techniques that provide a quantitative measure of a physical parameter (i.e., T_1_ or T_2_ mapping). In this respect, T_1_w/T_2_w is not a mapping technique as it is not quantitative (i.e., its value can be influenced by imaging sequence parameters, differences in head positioning during imaging, differences in signal scaling between the T_1_w and T_2_w acquisitions, and altered through the signal normalization step performed during processing (Glasser and Van Essen, 2011)); and it does not reflect an intrinsic tissue property (Shafee et al., 2014). Without strict normalization between the T_1_w and T_2_w acquisitions, the resultant ratio may be arbitrarily scaled between individuals, limiting its utility in comparison or association studies. Finally, T_1_w/T_2_w may not be applicable to non-cortex investigations (i.e., white matter or deep gray matter), or in non-neurotypical investigations, in which non-myelin pathology may alter the T_1_ or T_2_ signal. In contrast quantitative T_1_ and MWF mapping have lengthy histories in MRI investigations of normal and atypical brain and CNS tissue, though this is the first investigation of cortical MWF imaging.

## Conclusion

In this work, we examined intracortical myelination across early childhood using quantitative T_1_ and MWF imaging for the first time. We found that cortical MWF increased, and T_1_ decreased, logarithmically between the ages of 1 and 5 years. We also found qualitative agreement between regions exhibiting early (somatosensory, motor, and visual cortices) and late (frontal and temporal cortices) myelination and prior established histological patterns. Results lay the foundation for future studies examining the relationship(s) between measures of cortical maturation and cognitive and behavioral functioning, deviations in atypical development or disease, or the relationship between cortical and white matter maturation.

## Conflict of interest

The authors declare no competing financial interests.

## Figures and Tables

**Table 1:** Participant demographic information.

Gender	Male (*n*)	110
	Female (*n*)	105
Racial background	Caucasian (*n*)	156
	African American (*n*)	20
	Asian (*n*)	6
	Mixed race (*n*)	33
Mean age (days)	1051 ± 517	
Age range (days)	363 - 2198	
Mean gestation (weeks)	39 ± 3.2	
Mean birth weight (lbs)	7.6 ± 1.2	
Mean maternal SES	5.6 ± 1.7	

**Table 2:** Age-optimized imaging protocols.

		12–16 Months	16–28 Months	28–60 Months
	Field of view (cm)	17 × 17 × 14.4	18 × 18 × 15	20 × 20 × 15
SPGR	TE/TR (ms)	5.9 ms/12 ms	5.4 ms/12 ms	5.2 ms/11 ms
	Flip angles (degrees)	2, 3, 4, 5, 7, 9, 11, 14	2, 3, 4, 5, 7, 9, 11, 14	2, 3, 4, 5, 7, 9, 12, 16
	Bandwidth (Hz/pixel)	350	350	350
	Image matrix	96 × 96 × 80	104 × 104 × 84	112 × 112 × 84
IR-SPGR	TI/TE/TR (ms)	(600, 900) ms/5.9 ms/12 ms	(500, 850) ms/5.4 ms/12 ms	(500, 800) ms/5.2 ms/11 ms
	Flip angle (degrees)	5	5	5
	Image matrix	96 × 96 × 40	108 × 104 × 42	112 × 112 × 42
bSSFP	TE/TR (ms)	5.1 ms/10.2 ms	5 ms/10 ms	4.4 ms/9.8 ms
	Flip angles (degrees)	9, 14, 20, 27, 34, 41, 56, 70	9, 14, 20, 27, 34, 41, 56, 70	9, 14, 20, 27, 34, 41, 56, 70
	Bandwidth (Hz/pixel)	350	350	350
	Image matrix	96 × 96 × 80	104 × 104 × 84	112 × 112 × 84
High resolution IR-SPGR	Field of view (cm)	17 × 17 × 14.4	18 × 18 × 15	20 × 20 × 15
	TI/TE/TR (ms)	950 ms/6.9 ms/16 ms	950 ms/6.9 ms/16 ms	950 ms/6.9 ms/16 ms
	Flip angle (degrees)	5	5	5
	Image matrix	144 × 144 × 116	144 × 144 × 124	160 × 160 × 124

**Table 3:** Parsimony analysis of different functions that describe the change of cortical T_1_ and MWF with age. Values denoted by bold type signify the lowest BIC value, corresponding to model found to best describe the observed trajectories.

Cortical brain region	MWF			T1		
	Logarithmic	Linear	Quadratic	Logarithmic	Linear	Quadratic
Inferior temporal (left)	**5.26**	5.34	8.85	**− 12.79**	− 12.66	− 9.05
Inferior temporal (right)	**5.36**	5.44	8.95	**− 12.78**	− 12.68	− 9.09
Occipital pole (left)	**5.73**	5.78	9.28	− 12.11	**− 12.36**	− 8.81
Occipital pole (right)	**5.67**	5.74	9.2	**− 12.53**	− 12.45	− 8.93
Lingual gyrus (left)	**5.67**	5.71	9.22	**− 12.69**	− 12.70	− 9.14
Lingual gyrus (right)	**5.7**	5.73	9.21	**− 12.71**	− 12.69	− 9.18
Middle temporal gyrus (left)	**5.21**	5.28	8.84	**− 12.78**	− 12.67	− 9.06
Middle temporal gyrus (right)	**5.41**	5.46	8.93	**− 12.74**	− 12.67	− 9.17
Cingulate gyrus (left)	**5.56**	5.63	9.22	**− 12.93**	− 12.89	− 9.29
Cingulate gyrus (right)	**5.67**	5.74	9.27	**− 12.76**	− 12.72	− 9.12
Postcentral gyrus (left)	**5.55**	5.59	9.11	**− 12.79**	− 12.75	− 9.24
Postcentral gyrus (right	**5.59**	5.62	9.11	**− 12.86**	− 12.81	− 9.29
Precentral gyrus (left)	**5.58**	5.63	9.13	**− 12.81**	− 12.77	− 9.25
Precentral gyrus (right)	**5.74**	5.78	9.25	**− 12.75**	− 12.71	− 9.25
Inferior frontal gyrus (left)	**5.48**	5.56	9.04	**− 12.82**	− 12.72	− 9.18
Inferior frontal gyrus (right)	**5.52**	5.55	9.02	**− 12.87**	− 12.78	− 9.30
Superior frontal gyrus (left)	**5.72**	5.79	9.28	**− 12.85**	− 12.79	− 9.31
Superior frontal gyrus (right)	**5.66**	5.76	9.26	**− 13.00**	− 12.91	− 9.34
Temporal pole (left)	**5.49**	5.52	0.01	**− 13.25**	− 13.19	− 9.67
Temporal pole (right)	**5.31**	5.33	8.82	**− 13.44**	− 13.39	− 9.89
